# Prevalence of food insecurity and its association with depressive and anxiety symptoms in older adults during the COVID-19 pandemic in Mexico: A secondary analysis of ENCOVID-19 survey

**DOI:** 10.3389/fmed.2023.1110584

**Published:** 2023-03-13

**Authors:** De la Vega Martínez Alán, Rosas-Carrasco Oscar, Gaitán-Rossi Pablo, Ancira-Moreno Mónica, López-Teros Miriam

**Affiliations:** ^1^Health Department, Universidad Iberoamericana, Lomas de Santa Fe, Mexico City, Mexico; ^2^Research Institute for Equitable Development, EQUIDE, Universidad Iberoamericana, Mexico City, Mexico

**Keywords:** COVID-19, depression, anxiety, food insecurity, older adults

## Abstract

**Introduction:**

COVID-19 infection has caused high rates of morbi-mortality in older adults (OAs). In addition, conditions such as depression, anxiety, unemployment, and poverty frequently contribute to this population being at higher risk of food insecurity (FI) during the COVID-19 pandemic.

**Objective:**

This study aimed to analyze the prevalence of FI and its association with depressive and anxiety symptoms in Mexican OAs during the COVID-19 pandemic.

**Methods:**

This study involved a secondary analysis of the National Survey on the Effects of COVID-19 on the Wellbeing of Mexican Households (ENCOVID-19), a series of cross-sectional telephone surveys conducted between April and October 2020. The OA subsample was 1,065. FI was measured by using the Latin American and Caribbean Food Security Scale (ELCSA), and depression and anxiety symptoms were measured by using the Depression Scale of the Center for Epidemiological Studies (CESD-7) and the Generalized Anxiety Disorder Scale (GAD-2), respectively. Socioeconomic status (SES), occupation, schooling, and pension were also evaluated. ANOVA was used to compare the variables between the different FI groups, and logistic regression was used to analyze the risk between FI and the anxiety and depression variables.

**Results:**

The mean age of the participants was 67.31 ± 6.4 years, and FI was classified as mild, moderate, and severe, with prevalences of 38.6, 15.04, and 8.16%, respectively. Overall, 28.01% of the OAs presented symptoms of anxiety and 39.09% of depression. In the comparison between groups, a higher prevalence of depressive symptoms was observed with a higher degree of FI, with 65.75% in moderate-to-severe, 10.39% in mild, and 9.40% without FI, *p* ≤ 0.000. Regarding anxiety symptoms, 48% of the OAs showed moderate-to-severe, 30.05% showed mild, and 15.38% were without FI, *p* ≤ 0.000. Using multiple logistic regression, an OR of 5.50 (95% CI 2.74–11.04) was observed for depressive symptoms when moderate-to-severe FI is present. In the case of the risk of anxiety symptoms, it was significant in all degrees of FI, in mild (OR = 2.43, 95% CI 1.66–3.59) and in moderate-to-severe (OR = 5.32, 95% CI 3.45–8.19).

**Conclusion:**

There is a high prevalence of FI in Mexican OAs during the COVID-19 pandemic. FI increases the risk of other conditions such as depression and anxiety. It is important to design and implement programs aimed at OAs with these conditions to reduce or prevent FI.

## Introduction

The pandemic caused by the COVID-19 virus has caused the death of almost 6 million people in the world until February 2022 (1). Several studies have shown that age is an important predictor of adverse outcomes among patients with COVID-19 (2–4). A study published in Mexico in 2021 showed that the main predictors of severity and mortality in 220,804 confirmed cases of COVID-19 were age (adults 60 years of age or older) and high social lag indexes (2). In addition to comorbidities, high social lag indexes were identified as a predictor of mortality from COVID-19 adjusted for age and sex (HR 1.13, 95% CI 1.054–1.21).

In many countries of the world, social determinants of health have been observed to influence morbidity and mortality from COVID-19. These include poverty, physical environment, race, or ethnicity (5). OAs are usually a population with multiple risk factors such as chronic morbidity, lack of social support, living without company, and presenting greater effects on mental health such as anxiety or depression, reaching a range of 8.3 to 49.7% and 14.6 to 47.2%, respectively, according to a review by Sepulveda-Loyola et al. that included 42 articles with a total sample of 20,069 OAs from Asia, Europe, and America (6).

Gaitán-Rossi et al. through data from the National Survey on the Effects of COVID-19 on the Wellbeing of Mexican Households (ENCOVID-19) and the National Health and Nutrition Survey (ENSANUT 2018) in people over 18 years of age showed that the pandemic was also associated with a reduction in food security (households that did not report concerns or difficulties in accessing food), decreasing from 38.9% in 2018 to 24.9% in 2020 in Mexican households. At the lowest level of socioeconomic status, moderate-to-severe FI reached its highest prevalence, with 28.9 and 20.9%, respectively. Anxiety was also associated with higher FI scores; for example, 57.1% of people living with severe FI reported symptoms of anxiety (7).

Pourmotabbed et al. in a meta-analysis included 19 studies with 372,143 participants from 10 countries. The results showed that there was a positive relationship between FI and the risk of depression (OR = 1.40; 95% CI: 1.30–1.58) and stress (OR = 1.34; 95% CI: 1.24–1.44), but not anxiety. A subgroup analysis by age showed that adults older than 65 years had a higher risk of depression (OR = 175; 95% CI: 120, 256) than younger participants (OR = 1.34; CI 95%: 1.20–1.50). This study shows the relationship between FI and mental health status, such as depression and anxiety, and as previously stated, these conditions increased during the COVID-19 pandemic (8).

It is important to know the effects of the pandemic on the Mexican population, mainly in vulnerable populations such as OAs in a situation of poverty, high social lag indexes, greater comorbidity, and FI. There are still few published studies on FI in OAs in Mexico during the pandemic and the factors that could be associated with it. Therefore, the objective of this study is to analyze the prevalence of FI and its association with depressive and anxiety symptoms in Mexican OAs during the COVID-19 pandemic.

## Methods

### Study population and data sources

Secondary analysis of ENCOVID-19, which is a series of cross-sectional telephone surveys, is a national representative of people over 18 years of age who have a mobile phone. This survey provides data in four main domains: work, income, mental health, and food security, and began in April 2020 and continued monthly until October 2021, after which it was extended in frequency (9, 10).

The monthly surveys were compiled with probabilistic samples of mobile phone numbers using the national numbering plan, which is publicly available, as a sampling frame. To correct for slight deviations in the demographic composition of the ENCOVID-19 sample, post-stratification sample weights were used. Weights were calculated using data from the 2015 INEGI census to adjust the sample for geographic distribution (state), gender, age, and socioeconomic status (SES) (11).

For the present study, the rounds from April to October 2020 were used, including only people aged 60 years or older and who had data from the FI, depression, anxiety, and scales. A final sample of 1,065 participants was obtained.

### Measurements

Household FI was measured with the eight-item adult version of the Latin American and Caribbean Food Security Scale (ELCSA) (12), an instrument that has been validated for its use in Mexico (13). The adapted version of the scale (telephone) was used, which proved to be reliable and valid, and the alpha coefficients in the April, May, and June rounds varied between 0.87 and 0.89. Correlations between items were above the cutoff point of 0.60 in all surveys. Furthermore, the Rasch models showed that the high reliability of the telephone version of the scale was comparable to the face-to-face application in ENSANUT 2018 (10).

The ELCSA asks if, in the last 3 months, due to lack of money or other resources, the respondent or any other adult in the household: (i) worried that they might run out of food (worried); (ii) they were unable to eat healthy and balanced and nutritious (healthy) food; (iii) ate only a few types of food (few foods); (iv) skipped breakfast, lunch, or dinner (omitted); (v) ate less than he thought he should have eaten (without eating); (vi) the food is over (it is over); (vii) were hungry but did not eat (hungry); and (viii) went without eating for a whole day. Responses to all items are dichotomous (Yes/No).

After calculating the total summative score for the eight items, FI was classified into four levels: food secure (total score = 0), mildly food insecure (1–3), moderately food insecure (4–6), and food insecure severe (7, 8). For purposes of regression analysis, food insecurity was coded as no FI (0), mild FI (1), and moderate-to-severe FI (2). This is due to the number of severe OAs FI, which was only 75, so it was decided to join these two categories.

Anxiety symptoms. Anxiety was measured with the two-item generalized anxiety disorder scale (GAD-2) (14–16) which asks about how often the respondent felt during the last 2 weeks: (i) nervous, anxious, or borderline and (ii) not being able to stop or control the worry. Response options are “Never”; “several days”; “More than half the days”; and “almost daily.” The scale was validated in Spanish with the neuropsychiatric interview and was found to be reliable (alpha = 0.93) and with predictive validity with a sensitivity of 0.91, a specificity of 0.85, a positive predictive value of 86.6%, a negative predictive value of 91%, and an area under the curve of 0.937 (14–16).

Depression symptoms. The depression scale of the Center for Epidemiological Studies, abbreviated version (CESD-7) (17) was used. It consists of seven items that indicate the probable presence of depressive symptoms during the last week in which they were presented: (i) rarely or never (less than 1 day), (ii) rarely or sometimes (1–2 days), (iii) a considerable number of times (3–4 days), and (iv) all or most of the time (5–7 days). It has a minimum score of 0 and a maximum of 21 points, without symptoms <5 points and with the presence of depressive symptoms ≥5 points.

Socioeconomic status. The SES of the household was measured with the Mexican Association of Market Intelligence and Opinion Agencies (AMAI) index (18). It combines six indicators from the National Household Income and Expenditure Survey (2): (i) educational level of the household head; (ii) number of complete bathrooms; (iii) number of cars or vans; (iv) have an internet connection; (v) number of household members 14 years or older who are working; and (vi) number of bedrooms. Based on a summative score and standard cutoff points, the socioeconomic level is classified into seven mutually exclusive categories, ranging from “A/B” to “E,” where E represents the lowest value.

Other sociodemographic variables were also included, such as sex (male and female), age (years), level of schooling (basic (≤6 years), upper secondary, higher and postgraduate (>6 years), and no education (0 years)), occupation (active or not economically), welfare pension, and consumption of food groups (fresh fruits, vegetables, milk, eggs, meat, and beans), where the number of servings consumed per day is asked.

### Statistical analysis

A descriptive analysis of the characteristics of the population, presented with means±SD and absolute and relative frequencies, was carried out. Similarly, an ANOVA was performed to compare the study variables between the FI categories. In a third analysis, logistic regression models were adjusted according to the variables that were significant in the ANOVA and previously reported in the literature associated with FI, depression, and anxiety (sex, age, SES, and schooling) to identify the variables associated with terms of measures of association with odds ratios (ORs). Statistical power was calculated based on the prevalence of depression by FI level, giving a power greater than 80. The models were evaluated checking that there was no collinearity or interaction. Statistical significance was verified through the construction of 95% confidence intervals (95% CI). The statistical software used was Stata/SE, version 15.0 (Stata Corp., TX, United States).

## Results

The average age of the participants was 67.31 ± 6.4 years (60–99), 47.75% were women, and most of the participants had basic level education (≤6 years) (54.14%). The prevalence of FI in mild, moderate, and severe was 38.56, 15.04, and 8.16%, respectively, and 38.24% with food security. In relation to the consumption of different food groups, 23.79% reported that they stopped consuming fresh fruits, 21.52% vegetables, 32.79% meats, 24.12% dairy products, 12.66% eggs, and 6.11% beans. In relation to anxiety symptoms, a prevalence of 25.52 and 39.02% of depression symptoms was shown. Regarding the economic variables, 65.9% were within the economically inactive population, and when the participants were classified by SES, the levels with the highest prevalence were D and E (45.92%) and C, C−, and D+ (38.59%) (see Table 1).

**Table 1:** Description of the characteristics of the study population.

Characteristics of participants	Means ± SD or n (%)	*N*
**Sociodemographic**		
Age	67.31 ± 6.4	1,065
Gender (Women)	521 (47.75%)	511
(Men)	570 (52.25%)	554
Schooling (No education)	43 (7.73%)	556
(≤ 6 years)	301 (54.14%)	
(> 6 years)	212 (38.13%)	
**Food insecurity**		
Food insecurity scale (ELCSA-8)	2.01 ± 2.07	918
ELCSA with food safety	351 (38.24%)	
ELCSA mild FI	356 (38.56%)	
ELCSA moderate FI	136 (15.04%)	
ELCSA severe FI	75 (8.16%)	
Food consumption		
(Fruit)	3.03 ± 1.09	720
(Vegetable)	3.24 ± 2.0	
(Meat or egg)	3.67 ± 2.5	
(Milk and dairy products)	3.68 ± 2.9	
Less food consumption		
(Fresh fruit)	113 (23.79%)	473
(Vegetables)	61 (21.52%)	
(Meats)	75 (32.79%)	
(Dairy products)	55 (24.12%)	
(Eggs)	29 (12.66%)	
(Beans)	14 (6.11%)	
**Mental health**		
Anxiety symptoms (GAD-2 ≥ 3)	306 (25.52)	1,056
Depressive symptoms (CESD-7 ≥ 5)	119 (39.02)	303
Economy and occupation		
Occupation (Economically active)	160 (32.19)	497
(Economically inactive)	236 (65.9)	
Pension	272 (36.41)	272
SES		
1 (A/B and C+)	165(15.49)	1,065
2 (C, C− and D +)	411(38.59)	
3 (D and E)	489 (45.92)	

Values expressed as mean ± SD and/or n (%). ELCSA, the Latin American and Caribbean Food Security Scale; FI, Food insecurity; SES, Socioeconomic status; GAD-2, Generalized Anxiety Scale; CESD-7, Center for Epidemiological Studies.

A/B (Households in which the head of the family has professional education and has fixed internet at home. It is the level that invests the most in education and less in spending on food).

C+ (Most households at this level have at least one vehicle, 93% have access to fixed internet, and they spend just under a third on food purchases).

C (Most of the households at this level, the head, have education higher than primary school, 77% have a fixed internet connection, and allocate 35% of spending to food and 7% to education).

C− (Nearly three out of four households (74%) at this level have a head of household with higher than primary education. Just over half (52%) have a fixed internet connection at home. In relation to spending, 38% is dedicated to food and spending on transport and communication reaches 24%).

D+ (In just over 6 out of 10 households at this level (62%), the head of the household has higher than primary education. Only 22% of households have a fixed internet connection at home. Expenditure on food increases to 42% and spending on education is 7%).

D (In 56% of households at this level, the head of the household has education up to primary school. Internet access at home in these households is very low, only 4%. Close to half of the expenditure (46%) is devoted to food and only 16% to transportation and communication).

E (The vast majority of households at this level (95%) are headed by the head of the family with education up to primary school. Homeownership of fixed internet is practically zero (0.2%). Just over half of the household expenditure (52%) is used for food and only 11% is used for transportation and communication, a percentage similar to that used for housing).

In the comparison of the variables between the degrees of (in) food security, a higher prevalence of depression symptoms was observed at a higher degree of FI, 65.75% in moderate-to-severe FA, 10.39% in mild FI, and 9.40% without FI, *p* = 0.000. Regarding anxiety symptoms, 48% showed moderate-to-severe FI, 30.05% in mild FI, and 15.38% without FI, *p* = 0.000. Regarding the socioeconomic level, there was a higher prevalence of moderate-to-severe FI in the lowest status of SES, level D with 51.54% in severe FI, 40.84% in moderate FI, 37.91% in mild FI, and 25.48% without FI, level E with 19.49% in severe FI, 19.71% in moderate FI, 10.16% in mild FI, and 3.32% without FI (*p* = 0.000) (see Table 2).

**Table 2:** Comparison of the variables by the level of food insecurity.

Characteristics of participants	Food safety 351 n (%)	Mild FI 356 n (%)	Moderate-to-severe FI 211 n (%)	*p-*value
Gender (Women)	136 (38.75)	185 (51.97)	124 (58.77)	**0.000**
(Men)	215 (61.25)	171 (48.03)	87 (41.23)	
Schooling (No education)	7 (1.99)	14 (3.93)	19 (14.84)	**0.000**
(≤ 6 years)	88 (25.07)	122 (34.26)	84 (65.63)	
(> 6 years)	209 (59.54)	70 (19.66)	25 (19.53)	
Anxiety symptoms (GAD-2 ≥ 3 points)	54 (15.38)	107 (30.05)	100 (48.08)	**0.000**
Depression symptoms (CESD-7 ≥ 5 points)	33 (9.40)	37 (10.39)	48 (65.75)	**0.000**
Occupation (Economically active)	84 (39.44)	70 (32.86)	59 (27.70)	0.078
(Economically inactive)	118 (38.06)	129 (41.61)	63 (20.32)	
Pension (Yes)	85 (39.53)	93 (43.36)	37 (27.82)	0.0586
Socioeconomic status				
1 (A/B and C+)	38 (69.09)	14 (25.46)	9 (4.27)	**0.000**
2 (C, C− and D +)	60 (65.22)	26 (28.26)	58 (27.49)	
3 (D and E)	61 (57.55)	33 (31.33%)	144 (68.25)	

FI, Food insecurity. Bold values refers to the fact that they were significant values, however, it is suggested that it is quite bold.

In the final adjusted regression model, a significant association was observed for depressive symptoms with moderate-to-severe FI (OR = 5.50, 95% CI 2.74–11.04). In the case of the risk of anxiety symptoms, it was significant in all degrees of FI, in mild FI, an OR of 2.43 (95% CI 1.66–3.59) was observed, and in moderate-to-severe FI, an OR of 5.32 (95% CI 3.45–0.199) was observed.

## Discussion

The objective of this study was to analyze the prevalence of FI and its association with depression and anxiety symptoms in OAs in Mexico. With the results, we can observe that more than 60% of the participants are under some degree of FI, these data were similar to those reported by the different ENCOVID-19 surveys, for example, Gaitán-Rossi et al. reported that food security was 24.9% in Mexican households where a child lived, that is, 75% had an FI degree (7). Ponce-Alcala et al. according to data from the ENSANUT MC (2016) reported in 5456 adults aged 20 to 59 years that 70.8% had some degree of food insecurity at home (19).

In relation to the prevalences reported in other countries during the pandemic, Giacoman et al. through a longitudinal study based on two population-based surveys in Chile (CASEN 2017 and COVID 2020) found that FI levels went up significantly (*p* < 0.001) between 2017 (30%) and 2020 (49%) mainly in those with economically dependent people (that is, children, adolescents, and older adults). In this last population group, it was found that mild FI went from 12.7% in 2017 to 16.3% in 2020 and moderate-to-severe FI from 14 to 20.6% (20).

In the present study, it was shown that during the COVID-19 pandemic, at a higher degree of FI, there is a greater risk that OAs present anxiety and depression symptoms. This can be explained given the situation of the OAs since they were the ones who were in the greatest confinement due to their high risk of morbidity and mortality. In addition to these adverse effects associated with the pandemic, there was an increase in those OAs with the highest rate of social backwardness (3).

Few studies have analyzed the impact that the pandemic had on the mental health of OAs. Gaitán-Rossi et al. also found that anxiety was associated with higher FI scores during the pandemic, for example, symptoms of anxiety reported in people living in households with FI were 19.3% while in people living in households with severe FI were 57.1% (14). Sepúlveda-Loyola et al. (21) showed through a review that included 20,069 OAs, from Asia, Europe, and America during isolation due to the pandemic, presented a high prevalence of anxiety and depression, with a range of 8.3 to 49.7% and 14.6 to 47.2%, respectively. These results confirm the expected psychoemotional impact and the complex syndemic interaction of mental health and the FI experience during the pandemic (Table 3).

**Table 3:** Logistic regression on the association between food insecurity and depression symptoms.

Depression symptoms								
	No adjustment				Adjusted			
	OR	CI 95%		*P*	OR	CI 95%		*P*
FI mild	1.67	0.92	8.40	0.092	1.67	0.92	3.05	0.092
FI moderate-to-severe SES	**9.34**	3.09	28.23	**0.000**	**5.50**	2.74	11.04	0.000
2 (C, C− and D +)	1.09	0.31	3.78	0.886	**0.98**	0.40	2.35	0.967
3 (D and E)	1.92	0.55	6.69	0.304	0.640	0.19	2.16	0.479
**Schooling**								
≤6 years	1.14	0.47	2.77	0.757	1.36	0.49	3.80	0.570
>6 years	0.50	0.19	1.25	0.141	0.64	0.19	2.16	0.479
Woman	1.41	0.85	2.52	0.178	1.52	0.91	2.53	0.107
Age, years	0.97	0.35	5.08	0.255	0.97	0.94	1.01	0.292

FI, Food insecurity; SES, Socioeconomic status.Bold values refers to the fact that they were significant values, however, it is suggested that it is quite bold.

Moreover, in the descriptive analysis, we can observe that 20% of the population reported having stopped consuming fruits, vegetables, and dairy products and more than 30% reported having stopped eating meat, the latter being higher than that reported by Federik et al. (22), who reported less meat consumption in only 11.5% of young adults during the pandemic (Table 4).

**Table 4:** Logistic regression on the association between food insecurity and anxiety symptoms.

Anxiety symptoms								
	No adjustment				Adjusted			
	OR	CI 95%		*p*	OR	CI 95%		*p*
FI mild	2.437	1.66	3.59	**0.008**	**2.43**	1.66	3.59	**0.000**
FI moderate-to-severe SES	5.323	3.45	8.19	**0.000**	**5.32**	3.45	8.19	**0.000**
2 (C, C− and D +)	0.961	0.44	2.08	0.922	0.88	0.55	1.41	0.609
3 (D and E)	0.685	0.28	1.67	0.407	0.67	0.49	1.09	0.115
**Schooling**								
≤6 years	0.754	0.32	1.79	0.376	0.70	0.32	1.52	0.376
>6 years	0.963	0.27	2.07	0.586	0.70	0.24	2.07	0.456
Woman	**1.82**	1.25	3.45	**0.030**	1.60	1.18	2.18	**0.020**
Age,years	1.850	1.02	1.35	0.560	0.99	0.96	1.01	0.421

FI, Food insecurity; SES, Socioeconomic status.Bold values refers to the fact that they were significant values, however, it is suggested that it is quite bold.

The present study has the strength of having used the ENCOVID-19 data source, which has a representative sample of OAs from the 32 states of the Mexican Republic and the survey was carried out month-by-month. However, a limitation of ENCOVID-19 was the insufficient inclusion of people living in rural and isolated locations due to lower mobile phone coverage (9, 10).

Another variable studied was socioeconomic status, which was measured through a reliable asset-based scale suitable for implementation in brief telephone surveys. It has previously been shown, in face-to-face interviews, to be highly associated with income deciles across all states in Mexico and across localities with different population sizes (13). One limitation, however, is that this scale cannot capture changes in economic circumstances and only reflects pre-pandemic SES. However, in the present analysis, there was a higher prevalence of severe FI in households with lower levels of SES and it was associated with a higher risk of FI.

For future analyses, it is important to monitor the interaction of these factors (depression, anxiety, and SES) over time on the effects of FI and health in the older population during the pandemic. Furthermore, comparing these associations with pre-pandemic databases, we were able to measure the impact and incorporate other variables such as functional status, nutritional status, such as diet quality, and anthropometric and health data, such as comorbidity, and access to health services.

## Conclusion

There is a high prevalence of food insecurity during the COVID-19 pandemic, occurring in particularly vulnerable populations, such as older adults, in whom being food insecure has a higher risk of anxiety and depression symptoms. Interventions to increase access to healthy foods, especially among minorities and low-income people, and mitigate the socio-emotional effects are crucial to alleviating the economic stress of this pandemic.

## Data availability statement

The raw data supporting the conclusions of this article will be made available by the authors, without undue reservation.

## Ethics statement

The study was reviewed and approved by the Universidad Iberoamericana Research Ethics Committee (CONBIOETHICS-09 —CEI-008-2016060). Verbal Informed consent was obtained from all participants.

## Author contributions

DA contributed to data and analysis, collection and manuscript writing. L-TM contributed to statistic analysis and manuscript writing. R-CO collaborated in statistic analysis and manuscript review. G-RP contributed to database and manuscript review. A-MM manuscript review. All authors contributed to the article and approved the submitted version.

## Funding

This article was produced with the support of the Research Institute for Equitable Development with ENCOVID-19 funds, EQUIDE and Department of Health, University Iberoamericana, Mexico, Mexico City.

## Conflict of interest

The authors declare that the research was conducted in the absence of any commercial or financial relationships that could be construed as a potential conflict of interest.

The handling editor MA-B declared a past collaboration with the author L-TM.

## Publisher’s note

All claims expressed in this article are solely those of the authors and do not necessarily represent those of their affiliated organizations, or those of the publisher, the editors and the reviewers. Any product that may be evaluated in this article, or claim that may be made by its manufacturer, is not guaranteed or endorsed by the publisher.
